# The added value of using the HEPA PAT for physical activity policy monitoring: a four-country comparison

**DOI:** 10.1186/s12961-021-00681-6

**Published:** 2021-02-15

**Authors:** Peter Gelius, Sven Messing, Sarah Forberger, Jeroen Lakerveld, Fiona Mansergh, Wanda Wendel-Vos, Joanna Zukowska, Catherine Woods, the PEN Consortium

**Affiliations:** 1 Department of Sport Science and Sport, Friedrich-Alexander-Universität Erlangen-Nürnberg, Erlangen, Germany; 2 Leibniz Institute for Prevention Research and Epidemiology – BIPS, Bremen, Germany; 3 Department of Epidemiology and Data Science, Amsterdam Public Health Research Institute, Amsterdam UMC, VU University Medical Center, Amsterdam Public Health Research Institute, Amsterdam, The Netherlands; 4 Department of Health, Healthy Ireland, Dublin, Ireland; 5 National Institute for Public Health and the Environment, Bilthoven, The Netherlands; 6 Gdansk University of Technology, Gdańsk, Poland; 7 Physical Activity for Health Research Cluster, Health Research Institute, University of Limerick, Limerick, Ireland

**Keywords:** Physical activity, Policy, Monitoring, Ireland, Netherlands, Germany, Poland, HEPA PAT

## Abstract

**Background:**

Public policy is increasingly recognized as an important component of physical activity promotion. This paper reports on the current status of physical activity policy development and implementation in four European countries based on the Health-Enhancing Physical Activity Policy Audit Tool (HEPA PAT) developed by WHO. It compares the findings to previous studies and discusses the general utility of this tool and its unique features in relation to other instruments.

**Methods:**

The study was conducted as part of the Policy Evaluation Network (www.jpi-pen.eu) in Germany, Ireland, the Netherlands and Poland. Data collection built upon information obtained via the EU Physical Activity Monitoring Framework survey, additional desk research and expert opinion. Data analysis employed Howlett’s policy cycle framework to map and compare national physical activity policies in the four countries.

**Results:**

In all countries under study, policy agenda-setting is influenced by prevalence data from national health monitoring systems, and the sport and/or health sector takes the lead in policy formulation. Key policy documents were located mainly in the health sector but also in sport, urban design and transport. Physical activity programmes implemented to meet policy objectives usually cover a broad range of target groups, but currently only a small selection of major policies are evaluated for effectiveness. National experts made several suggestions to other countries wishing to establish physical activity policies, e.g. regarding cross-sectoral support and coordination, comprehensive national action plans, and monitoring/surveillance.

**Conclusions:**

This study provides a detailed overview of physical activity policies in the four countries. Results show that national governments are already very active in the field but that there is room for improvement in a number of areas, e.g. regarding the contribution of sectors beyond sport and health. Using the HEPA PAT simultaneously in four countries also showed that procedures and timelines have to be adapted to national contexts. Overall, the instrument can make an important contribution to understanding and informing physical activity policy, especially when used as an add-on to regular monitoring tools like the EU HEPA Monitoring Framework.

## Background

Noncommunicable diseases (NCDs) are a leading cause of global mortality, responsible for 40 million of the 56 million global deaths in 2015 [1]. Data from the WHO Global Health Observatory show that 80% of premature heart disease, stroke and diabetes could be prevented [1]. Insufficient levels of physical activity (PA) are considered to be a key behavioural determinant for underlying NCDs, responsible for around 5–6 million premature deaths per year worldwide [2, 3]. It is estimated that physical inactivity causes 6% of the burden of disease from coronary heart disease, 7% of type 2 diabetes, 10% of breast cancer and 10% of colon cancer [2]. Furthermore, studies have shown that regular PA is an effective strategy against at least 25 chronic medical conditions and can reduce the risk typically by 20–30% [4].

Besides specific “downstream” solutions like interventions to promote PA (such as structured exercise programmes or infrastructure design), public policy is increasingly recognized as an important “upstream” component of PA promotion, as policy actions to address lifestyle behaviours have the potential to influence health and well-being of an entire population [5]. In order to develop future country-level policies and international action plans, a thorough understanding of current national activities and contexts is key. However, our knowledge about the current status, implementation and effectiveness of policies that can promote PA in different countries is still very limited, and there is consequently no clear guidance on which policies governments should preferably use in different settings or under various preconditions.

In order to address this problem, there have recently been several activities to foster the collection of systematic, comparable data on national PA policies. This includes the development of toolkits such as the WHO Health-Enhancing Physical Activity (HEPA) Policy Audit Tool (PAT) [6, 7] that served as a basis for the Monitoring Framework for the EU Council Recommendation on HEPA Across Sectors [8], the WHO developed Monitoring Framework for its Global Action Plan on Physical Activity (GAPPA) [9] and the Comprehensive Analysis of Policy on Physical Activity (CAPPA) framework [10].

The following analysis of national PA policies is based on the HEPA PAT, a tool provided by WHO to facilitate a situational analysis within a country that involves relevant actors from public sectors, nongovernmental organizations, and academia, thus supporting national capacity building and informing decision-making. The tool can also be utilized as an instrument for collecting internationally comparable data on PA policies and their development [6]. The PAT was developed between 2009 and 2011 using a literature search of cross-country comparisons on PA policy, followed by a pilot study in seven countries to improve its usability and ensure its applicability across different contexts [6, 11].

This paper reports on the results of auditing PA promoting policies in Germany, Ireland, the Netherlands and Poland using the PAT. In addition, it provides information on the practical aspects of applying the tool in different national contexts, compares the results to findings from previous studies, and discusses the general utility of this tool and its added value when used in combination with other existing instruments.

## Methods

### Case selection and basic rationale

This study was conducted in Germany, Ireland, the Netherlands and Poland as part of the Policy Evaluation Network (PEN, www.jpi-pen.eu), a project consortium composed of 28 research institutions from seven European countries and New Zealand [12]. The four countries were selected as case examples from the total of eight PEN countries to ensure a level of variation regarding geography (Western, Central and Eastern Europe), population size (small, medium and large) and mode of government (centralist vs. federalist). Data were collected using Version 2 of the HEPA PAT [7], a standardized instrument to assess national policy approaches to PA. The PAT comes in the form of a questionnaire with 29 closed and open-ended questions to support standardized data collection. It is to be completed collaboratively by a team of national stakeholders from all relevant sectors (for instance health, sport, education, transport environment, and urban planning) under the supervision of a project team and a coordinator [7]. While similarities exist with other tools, namely the EU Monitoring Framework [6], the PAT intends to combine expert opinion with academic desk-based research to provide additional in-depth information on key indicators.

### Data collection

In all four countries, data collection was academia-driven and coordinated by the participating PEN scientists. These researchers formed a cross-national coordination team that started by dividing the questions of the PAT into three categories: Category I, questions that could be (partially) answered by referring to data recently collected from national governments during the 2018 round of the EU PA Monitoring Framework survey; Category II, questions that could be addressed by conducting desk research; and Category III, questions that required obtaining the opinion of experts. Table 1 presents an overview of the PAT’s questions and our own categorization, while Table 2 provides some examples for questions from the category “decision-making”.

**Table 1:** Overview of PAT questions and categorization of data collection and analysis

Position in policy cycle	PAT indicator		Main data source		
	Topic	No	Category I (2018 PA Focal Point survey)	Category II (desk research)	Category III (expert opinion poll)
Policy-making structures	Government structure	1a		√	
	Governance at subnational level	1b		√	
	Main government ministries	1c		√	
	Other important national organizations	1d		√	
Agenda-setting	Health surveillance or monitoring system	20	√	√	
	Influence of data on the prevalence of PA or sedentary behaviour on policy development	21a			√
	Influence of surveillance data on the national promotion of PA	21b			√
Policy formulation	Leadership for HEPA promotion at the national level	2	√		
	Leadership for HEPA promotion at the subnational level	3		√	√
	Cross-sectoral collaboration at the national level	4	√		
	Cross-sectoral collaboration at the subnational level	5		√	√
	Evidence-base of key policy documents	10		√	
	Usefulness of international documents	11			√
	National recommendations on PA and health	17a	√	√	
	National recommendations on reducing sedentary behaviour	17b	√	√	
	National goals (or national targets) for population prevalence of PA	18	√	√	
	Other goals and targets that directly or indirectly relate to PA	19	√	√	
Decision-making	Key past policy documents and past events	6		√	√
	Current key policy documents, legislation, strategies or action plans	7	√	√	√
	Use of a consultative process	8	√	√	
	Evidence of cross-referencing and alignment within and between policies	9		√	√
Policy implementation	National documents or guidelines to support implementation of HEPA activities at the subnational level	12		√	√
	Settings included for the delivery of specific HEPA actions	13	√	√	
	Populations targeted by specific HEPA actions	14	√	√	
	National communication strategy (using mass media)	15	√		
	Examples of large-scale programmes	16		√	√
	Funding at the national level	24a	√		
	Funding at the subnational level	24b		√	√
	Political commitment	25			√
	Network or system that links and/or supports professionals	26		√	
Evaluation	Health surveillance or monitoring system	20	√	√	
	Evaluation of national policies or action plans	22a	√		
	Evaluation at the subnational level	22b		√	√
	Economic evaluation of interventions or physical inactivity	23		√	
	Areas of greatest progress in national HEPA promotion	27a			√
	Biggest challenges in national HEPA promotion	27b			√
	Suggestions for other countries	28			√
Other	Further details or comments	29		√

**Table 2:** Examples of PAT questions for the decision-making category

Topic	No.	Question
Key past policy documents and past events	6	Please describe any key past policy documents and past events that have led to the current context of HEPA promotion in your country. This might include legislation or recent policy documents that are now technically out of date (e.g. a previous national HEPA policy that may or may not have been extended), previous landmark legislation, or other documents such as scientific reports. Key events might include political changes, position statements or scientific events that have shaped the HEPA agenda Please list the documents/events, provide a web link (where available), and indicate if an English version or summary is available in each case
Current key policy documents, legislation, strategies or action plans	7	Please provide details (title, timeframe, issuing body) of the current key policy documents, legislation, strategies or action plans in your country, which outline government (and, where applicable, NGO) intention to increase national levels of physical activity (see Glossary for definitions of these terms) Please list the documents according to sector and, where available, provide a web link, indicating whether an English version or summary is available. Please provide a brief description of the general content of each policy (about 100–250 words) Please mark in the right-hand column which are the most important documents for the HEPA agenda in your country and briefly explain why these documents are deemed important
Use of a consultative process	8	During the development of the most important policies/action plans listed in Question 7, was a consultative process used, involving relevant stakeholders? If yes, please briefly outline the steps of this consultation processes and which organizations were involved. Please also mention any challenges in recent years in engaging government ministries or other agencies through such processes
Evidence of cross-referencing and alignment within and between policies	9	In your appraisal of the policy documents listed in Question 7, is there evidence of cross-referencing and alignment within and between policies, with genuine connections between different policy areas, or do the policies present separate, sector-specific strategies without evidence of links and consistency across sectors and documents with relevant policy? For example: in the health sector, does a national obesity prevention strategy refer to an existing physical activity promotion plan, thus demonstrating an integrated overarching national approach to addressing physical activity? Does a transport policy recognize links with other policies that promote walking and cycling in the health sector (or sport field)? Does a sport promotion policy cross-reference HEPA promotion activities contained in a health promotion policy? If yes, please briefly explain and give examples of such cross-referencing. Please state which of the policy documents presented in Question 7 you are referring to

Data were collected between March and October 2019. All four countries followed the same six-step process, making slight adaptations where necessary to account for their specific country context: PEN project researchers (1) identified national stakeholders/experts to complete the PAT, (2) pre-filled data on Category I questions from the 2018 survey for the EU Monitoring Framework after obtaining consent from national Focal Points and WHO, (3) conducted desk research to identify and review all available policy documents, programmes and relevant activities from across multiple sectors to collect additional information on Category II indicators, (4) obtained verification of details and additional facts from select institutions and organizations where necessary, and (5) collected expert input on Category III indicators using workshops, interviews and/or online surveys. In a final step (6), the PEN team combined all information from the previous steps to fill out the PAT template for each of the four participating countries. In all countries, the PAT templates were filled out in English to facilitate a cross-country comparison.

### Data analysis

The primary aim of data analysis was to map PA policy at the national level and to identify similarities and differences between the four countries on key themes of interest. Inspired by a previous study of PAT data for a sample of seven countries [11], data analysis was performed using directed content analysis, i.e. the overarching categories of the PAT served as initial themes for the analysis. In order to structure the large amount of data for further analysis, we employed the stage model of the policy process originally introduced by Laswell [13], which divides policy-making into a series of distinct phases. This “heuristic” [14] was subsequently further developed into a policy cycle model [15] to better represent the complex system of drafting, implementing and assessing a policy. It assumes that the stages tend to repeat over and over again as policy is further developed and adapted to real-world circumstances. The approach has been applied to the field of health and health promotion by various authors [16–18]. In this audit of PA policies, the main benefit of applying the policy cycle model is the division of policy-making into different phases, which prompts policy-makers and stakeholders to think about the process in a systematic way when identifying actors, approaches and outputs for each step and also helps to structure the subsequent analysis. Table 1 shows how the 29 indicators of the HEPA PAT were structured according to the categories of the policy cycle developed by Howlett et al. [15].

As the answers to the open-ended questions in the PAT represent the consolidated opinion of all individuals and organizations involved, they were not coded in the way usually employed for qualitative interviews. Instead, the lead authors of the paper conducted a first screening of all four PATs on the basis of the policy cycle identifying key structures, actors, processes and/or outputs for each phase. Initial results were collated, fed back to the national PEN researchers for comments and clarification, and discussed with the entire research group to identify key results. These will be presented in the next section for each of the following categories: policy-making structures, agenda-setting, policy formulation, decision-making, policy implementation and policy evaluation.

## Results

### Policy-making structures

Results show that government structure varies substantially between the four countries: while three are unitary states with a strong central government, Germany has a federal system that warrants a close look at the regional-level to obtain a full picture of the status of policy implementation. In addition, ministry portfolios vary substantially across countries, which is also likely to impact PA policy-making. Notably, while all four countries have separate ministries for sport and health, Ireland has a single ministry with competences in three PA-related sectors: the Department of Transport, Tourism and Sport.

### Agenda-setting

All four countries reported the existence of a national system to monitor PA levels in the population: the “National Health Monitoring” in Germany [19]; the “Sports Monitor”, “Children’s Sport Participation and Physical Activity Study” and the “Healthy Ireland Survey” in Ireland [20–22]; the “National Health Interview Survey/Lifestyle monitor” in the Netherlands [23]; and the “National Talent Base” in Poland [24] are examples of monitoring systems operational in each country. The last of these is a publicly available tool to monitor physical fitness using somatic measurements, which had nearly 2 million entries in 2017.

The countries also take part in the EU’s European Health Interview Survey (EHIS) and the Eurobarometer surveys, which include information on PA and Sports at the population level [25, 26]. In addition, they participate in the EU’s policy Monitoring Framework for HEPA Across Sectors (2015, 2018), which also informed the completion of the PATs for the study at hand, and WHO Europe’s Health Behaviours in School-aged Children (HBSC) study [27, 28].

All four countries reported that data on the prevalence of PA or sedentary behaviour influenced policy development in their country. Such data were used as a basis for the development of policies, e.g. the National Physical Activity Plan “Get Ireland Active” (NPAP, Ireland, [29]), the National Sports Agreement (Netherlands, [28]), the Sport Development Programme 2020 (Poland, [30]) as well as the National Action Plan IN FORM and the National Recommendations for Physical Activity and Physical Activity Promotion (Germany, [31, 32]). Additionally, several countries mentioned the importance of prevalence data for policy monitoring, e.g. regarding the Sport and PA Close to Home Programme (Netherlands, [33]) and the NPAP (Ireland, [29]).

By contrast, only Ireland and Germany reported that surveillance data helped to advance the national promotion of PA in any other way. In Ireland, surveillance data are used by government ministers in their public speeches and as an evidence base for public health measures, programmes or infrastructure. Germany stated that surveillance data increased the awareness of PA promotion in politics, society and the media.

An overview of the identified policies in the phase of agenda-setting is provided in Table 3.

**Table 3:** Overview of policies in the phase of agenda-setting

	Germany	Ireland	Netherlands	Poland
National health surveillance or monitoring system	√	√	√	√
Participation in EU health surveillance and monitoring systems	√	√	√	√
Influence of prevalence data on policy development	√	√	√	√
Influence of surveillance data on the national promotion of PA	√	√	-	-

### Policy formulation

In Ireland, leadership for PA promotion at the national level is shared between the sport (Department of Transport, Tourism and Sport and its agency Sport Ireland) and health sectors (Department of Health and its subordinate agency, the Health Service Executive HSE). The same is true for the Netherlands, where leadership is shared between the Knowledge Centre for Sport, the National Institute for Public Health and the Environment RIVM, and the Netherlands Organization for Health Research and Development (ZonMw). By contrast, the Ministry of Sport and Tourism (Department of Sport for All) has the lead of national PA policy in Poland, while policy in Germany is coordinated from within the health sector, namely the Ministry of Health and its subordinate agencies (such as the Federal Centre for Health Education BZgA and the Public Health Institute RKI).

Ireland and Germany also reported the existence of cross-sectoral coordination mechanisms at the national level. In both cases, these are working groups overseeing the implementation of the national PA action plans (Ireland: NPAP Implementation Group; Germany: IN FORM HEPA Working Group). The German National Action Plan IN FORM, which addresses both healthy nutrition and physical activity, is itself a result of cross-sectoral coordination between the Ministry of Food and Agriculture and the Ministry of Health [31].

All countries reported having national targets for the population prevalence of physical activity. While Ireland aims to increase the proportion of people fulfilling the PA recommendations by 1% per annum for each target group (children, adults, older adults) [29, 34], the goal in the Netherlands is that 75% of the population adhere to the national PA guidelines by 2040, compared to 47% in 2017 [34]. Poland intends to lower the percentage of citizens who never do sports or who are not sufficiently physically active [30] and Germany formulated a more qualitative goal of aiming to achieve “visible” results by 2020 [31]. However, by adopting the WHO Global Action Plan for the Prevention and Control of NCDs 2013–2020 [35], which includes nine voluntary NCD targets, all four countries agreed to aim for a 10% reduction in prevalence of insufficient physical activity by 2025 [36].

Several “other” goals and targets that directly or indirectly relate to PA promotion are connected with sports, such as increasing the amount of people taking regular exercise (Ireland, [29]) and winning medals at the Olympic Games (Ireland, [34]; Netherlands, [37]). Other goals stem for example from the transport sector, such as a 10% reduction of the energy consumption in the traffic sector in Germany by 2020 [38].

In Ireland, Poland and Germany, experts were asked to rate the usefulness of international policy documents published by WHO, the EU, and other international organizations. The evidence suggests that WHO’s 2004 Global Strategy on Diet, Physical Activity and Health [39], as well as the EU’s 2013 Council Recommendation on HEPA Across Sectors [8] were considered to be of particular importance for national policy-making. By contrast, the impact of academia-driven key documents such as the Toronto Charter [40] and the Lancet series on PA seems to vary across countries [41].

An overview of the identified policies in the phase of policy formulation is provided in Table 4.

**Table 4:** Overview of policies in the phase of policy formulation

	Germany	Ireland	Netherlands	Poland
Leadership for PA promotion at the national level (sector)	Health	Shared	Shared	Sport
Cross-sectoral collaboration at the national level	√	√	–	–
National goals (or national targets) for population prevalence of PA	√	√	√	√
Other goals and targets that directly or indirectly relate to PA	√	√	√	√

### Decision-making

Table 5 presents an overview of the most relevant sectors covered by national PA legislation, policies or other relevant documents (such as recommendations) in the four countries. While all four countries reported PA policies, legislation and/or other documents in the fields of health, transport and environment/urban planning, there were differences in the areas of sport and education. These differences may be explained in part by countries’ specific political systems: the German constitution, for example, defines sport as part of cultural policy, which is the domain of the 16 state governments. As a consequence, the country has no national PA policy with a sports perspective. The same is true for education, which is also part of the competence of the states. Responses also indicate that documents such as national strategies, action plans and governmental interagency agreements appear to be the dominant document type in the four countries, while formal legislation only exists in the health sector.

**Table 5:** Presence of legislation, policy and action plans by sectors and country

Country	Health			Sport^a^			Education			Transport			Environment, urban planning		
	L	P	O	L	P	O	L	P	O	L	P	O	L	P	O
Germany	√	√	√	S	S		S	S			√			S	√
Ireland		√	√		√			√	√		√	√		√	
Netherlands		√			√						√			√	
Poland		√			√						√			√	

In Germany, some areas fall under the jurisdiction of the 16 state governments

*L* legislation, *P* policy, *O* other relevant documents

^a^This category refers to sport for all rather than only elite sport

In addition, all countries identified a number of key policy documents and indicated which of them could be considered to be the most important. A comparison of these documents shows that national recommendations are considered relevant (e.g. Germany, Ireland, [29, 32]) and that all countries reported key policy documents from the health sector. Almost all countries identified at least one key policy document in the sports sector (except Germany) and in the urban design or transport sector (except Poland). Poland was the only country that reported a key policy document for physical activity promotion from the social policy sector [42]. Most of these documents were published in the last decade.

For all countries, the desk research identified evidence of cross-referencing and alignment within and between policies. In Ireland and Germany, there is one central policy document to which all the other policy documents refer regarding physical activity promotion (the National Action Plan IN FORM for Germany [31] and the NPAP for Ireland [29]). By contrast, Poland and the Netherlands did not identify such a central policy document. While Poland reported intersections of documents regarding specific policies for physical activity promotion, the Netherlands identified several explicit cross-references between health, sport, education and active transport policies [43].

An overview of the identified policies in the phase of decision-making is provided in Table 6.

**Table 6:** Overview of policies in the phase of decision-making

	Germany	Ireland	Netherlands	Poland
Current key policy documents, legislation, strategies or action plans	√	√	√	√
Evidence of cross-referencing and alignment within and between policies	√	√	√	√

### Policy implementation

The following target groups are addressed by specific HEPA actions in all four countries: children/young people, older adults, clinical populations/chronic disease patients, people from low socioeconomic status and families. Other population groups—early years, workforce/employees, women, people with disabilities, sedentary/the least active, migrant populations and the general population—are also addressed, but only in three out of four countries each (Table 7).

**Table 7:** Population groups targeted by specific HEPA actions

	Germany	Ireland	Netherlands	Poland
Early years	√	√		√
Children/young people	√	√	√	√
Older adults	√	√	√	√
Workforce/employees	√	√	√	
Women	√	√		√
People with disabilities	√	√		√
Clinical populations/chronic disease patients	√	√	√	√
Sedentary/the least active	√	√		√
People from low socioeconomic status	√	√	√	√
Families	√	√	√	√
Migrant populations	√	√		√
General population	√	√	√	

Only Ireland reported to have a national communication strategy using mass media that aims to raise awareness and promote PA. This strategy resulted in two campaigns that linked PA to obesity (START campaign, [44]), healthy eating and mental well-being (Healthy Ireland Communications Campaign, [45]). In Germany, various PA-promoting activities are accompanied by separate awareness-raising measures, while Poland and the Netherlands stated that no national communication strategies are currently in place.

In each of the four countries, the delivery of PA-related policies or action plans is supported through funding at the national level. However, the variety of funding sources makes it difficult to determine the total amount of funding. Additionally, it is not always possible to distinguish funding intended to promote PA from other purposes, e.g. infrastructure improvement or environmental policy. Concrete examples for PA-related funding include the Irish Bike to Work Scheme [46], the Polish National Health Programme 2016–2020 [47] and the German National Action Plan IN FORM [31].

An overview of the identified policies in the phase of decision-making is provided in Table 8.

**Table 8:** Overview of policies in the phase of policy implementation

	Germany	Ireland	Netherlands	Poland
Populations targeted by specific HEPA actions	12/12	12/12	7/12	10/12
National communication strategy (using mass media)	Partially	√	–	–
Funding at the national level	√	√	√	√

### Policy evaluation

Completing the PAT also provided information regarding the evaluation of major policies. In Poland, the major policies contained dedicated plans for evaluating impacts. The two policies specifically mentioned were the Sport Development Programme 2020 [30] and the Regulation of the Council of Ministers concerning the National Health Programme for the years 2016–2020 [47]. Likewise, major Irish policies (the NPAP, the National Sports Policy, and the Healthy Ireland Framework) included evaluation components [29, 34, 48]. By contrast, evaluation is only included in part of the national policies in Germany and the Netherlands. In Germany, this applies to the National Action Plan IN FORM and the National Cycling Plan but not to the National PA Recommendations [31, 32, 38]. In the Netherlands, some policies such as the Sport and PA Close to Home Programme are evaluated [33, 49], while others such as Sports Without Boundaries are not [50, 51]. The National Sport Agreement states that the policy will be adjusted if national PA monitoring indicates that there is reason to do so [28].

In addition, the experts involved in answering Category III questions were asked to use their personal assessment of national PA policy to provide suggestions for other countries intending to set up their own HEPA policies. Table 9 provides a summary of key recommendations made by experts from all four countries, and provides an overview of the policies identified in the phase of evaluation.

**Table 9:** Overview of policies in the phase of evaluation, including suggestions for other countries

	Germany	Ireland	Netherlands	Poland
Evaluation of national policies or action plans	Partially	√	Partially	√
Suggestions for other countries	• Involve relevant stakeholders in policy development • Adopt a national action plan (cross-sectoral, evidence-based) • Use national recommendations as an approach to activate stakeholders	• Understand your starting point well • Have clear goals and key performance indicators (KPIs) • Establish high level, cross-government support—outside the usual actors • Be as creative as your resources will allow • Measure what you trial	• Enhance governmental support on development of local policy, strategies and networks • Tailor policy measures to target populations and settings • Enhance cooperation across sectors	• Launch a national HEPA focal point and an organisation coordinating HEPA activities • Develop a method for measuring HEPA for individual age groups • Develop and implement a national programme for HEPA promotion

## Discussion

We have attempted to support PA policy benchmarking efforts by shedding light on the current implementation of national PA policies in selected European countries, and by reflecting on the tools to do so. We believe that the results provide an interesting in-depth view of the status of PA policy, the range of potential policies, and important factors shaping national policy development in Europe. Regarding *policy-making structures*, results show some interesting variations across countries. Features such as the strong regional level of government in Germany suggest that the influence of such characteristics on national policy-making should be further investigated, and that additional data collection may be necessary to obtain a complete picture of PA policy-making in a country. While national health monitoring and PA prevalence data appear to play a similar role for policy *agenda-setting* in all countries, the different ways of organizing policy portfolios are again reflected in leadership for *policy formulation*. The two models found in our sample are leadership by either a single sector (sport in Poland, health in Germany) vs. shared leadership (health, sport and transport sector in Ireland and the Netherlands). The comparative analysis of national policy documents shows that *decision-making* seems to occur mainly with a health and sport perspective in mind, followed by the education, transport and environment/urban planning sectors. Interestingly, there is only one country (Poland) with a key PA policy document in the social policy sector. Also, most documents seem to come in the form of action plans or strategies, while legislation appears to be limited mostly to the health sector. Regarding *policy implementation*, there appears to be a good coverage of major population groups in the form of PA programmes and interventions in all countries. However, it is striking to note that the intersectoral character of PA promotion and the multitude of actors makes it virtually impossible even for national governments to obtain an overview of the total amount of funding invested in the field. More data would be highly desirable to inform future policy-making, but innovative approaches would be needed to enable this. Finally, the need for *policy evaluation* is recognized in all four countries, but our results seem to indicate that there is still room for improvement in this area, as by far not all major policies currently seem to have built-in evaluation mechanisms.

Our findings mostly confirm the results of previous studies using the PAT or the related EU HEPA Monitoring Framework. For example, in their comparative study employing the PAT in seven European countries, Bull et al. [11] also found that health, sport and education had supportive policies in place but that there was room for improvement in the transport and environment sector. Similarly, Breda et al. [52] concluded that PA policy in EU Member States was generally less developed in the areas of senior citizens, workplace health promotion and environment/urban planning/public safety.

As in previous studies [53], the approaches used to engage policy-makers and experts differed substantially between our four study countries. In general, however, our use of a research-led, standardized completion process was arguably more homogeneous than previous PAT completions, thus potentially allowing for better cross-country comparability. Conversely, owing to our strong focus on desk research to pre-fill the questionnaire, the capacity-building effects of completing the tool in a cross-sectoral expert group emphasized in the existing literature on the PAT [11, 53, 54] may not have been as pronounced in our project (possibly with the exception of Ireland, where several larger-scale stakeholder meetings were held to verify data).

Our results also provide important insights regarding the practical suitability of the HEPA PAT as a tool to collect policy data at the national level. We found that data collection is dependent on the availability of specific government officials and on functioning national expert networks. This meant that data collection procedures, stakeholder group composition, strategies to obtain expert opinion and feedback, and timelines varied substantially between countries. At various points in the process, the coordination group considered a stricter harmonization of procedures to potentially increase comparability of results but decided against it. A “one size fits all” approach would have led to severe loss of information in several countries as it was foreseeable that timelines would not be met, or important stakeholders might not respond to certain forms on engagement (e.g. workshops). At the same time, our experience shows the flexibility of the HEPA PAT to accommodate for national differences and points to its applicability in various contexts. Our experience with the PAT is mirrored by other studies that have used the tool, e.g. regarding difficulties with data collection on funding and subnational policies [52] or challenges completing the process within short timeframes [53].

An important question for both researchers and policy-makers is whether the considerable workload for completing the HEPA PAT is warranted by a corresponding added value over other existing tools for PA policy monitoring. This is particularly relevant for EU Member States, as 14 of its questions are also related to the EU HEPA Monitoring Framework, a tool that all Member States have agreed to complete every 3 years. In our view, two important arguments for using the HEPA PAT even in EU countries are the extra facts and details provided by its desk research element and the opportunity to include expert opinion. In this way, regular data collection via the Monitoring Framework could be supplemented by intermittent full-fledged surveys using the HEPA PAT. This seems a particularly sensible approach if one bears in mind that the HEPA PAT was an important inspiration for the development of the Monitoring Framework.

Other countries might benefit from this cross-country comparison in a number of ways. First of all, the study has identified relevant policies and policy-making approaches from Germany, Ireland, the Netherlands and Poland which could inspire policy-makers and stakeholders in countries with similar contexts (e.g. regarding population size and political structure). This also applies to the suggestions that were made by experts on the basis of their experience of PA policy-making in their respective country. A more general aspect highlighted by our research is the need for a comprehensive approach to PA policy-making that includes each phase of the policy cycle. Finally, the methodology employed may serve as a blueprint for future PA policy audits using the HEPA PAT and other policy monitoring tools.

Our study comes with a number of limitations, mostly related to the short timeline for data collection prescribed by the necessities of the overall project and the limited resources available for desk research. For example, it was not possible to obtain detailed information on subnational policy-making in Germany by making inquiries with responsible ministries and agencies of all 16 regional governments. Additionally, even though a standardized six-step process was applied, a certain risk of bias remains as adaptations to the country context had to be made: while some countries verified facts by approaching important stakeholders individually, others organized workshops to present the pre-filled PAT to a group of experts (or used a combination of both approaches). Likewise, the Irish expert group contained a larger number of ministry representatives, while there were more university researchers on the German expert group, potentially leading to different overall perspectives of the expert input (Category III questions). Furthermore, data collection took place in 2019, and new political decisions might have been made in the meantime. For Germany, the Netherlands and Poland, language issues may also have to be considered, as answers and questions had to be translated back and forth between English and the respective national language. Finally, this paper does not replace a detailed analysis of PA policy-making in each of the four countries. Such an analysis could provide additional and highly relevant insights but would be far beyond the scope of this paper.

## Conclusions

This study has helped provide a more detailed and up-to-date overview of PA policy-making in Ireland, the Netherlands, Germany and Poland. Results have shown that countries are already very active in the field but that there is room for improvement in a number of areas. An important implication for national policy-making is to try to further increase awareness for the issue in sectors beyond sport and health (such as education, transport, urban planning and tourism) and increase their contribution to PA policy. Another task is to create mechanisms to ensure that all future PA policies are evaluated with respect to their effectiveness.

With respect to policy monitoring, the experience of our study suggests that a research-driven, systematic approach to completing the PAT is highly compatible with other tools and existing frameworks. For EU countries in particular, a potential way towards regular and detailed PA policy monitoring could be to use the EU’s triennial survey on the HEPA Monitoring Framework as a basis to conduct intermittent, more in-depth monitoring exercises using a pre-filled PAT, which would provide policy-makers and practitioners within nations with valuable additional information. The relationships and networks established by the study at hand could be used for future updates, but political support at the national level and adequate, reliable resourcing would be needed to build a permanent monitoring mechanism.

Potential tasks for future research in this area include focusing further on subnational policy-making and identifying effective, parsimonious means to collect and handle all relevant data, especially in federalist states. Efforts could even be further extended by building on first efforts to establish a PAT for municipalities [54, 55]. In addition, research could turn its attention to the potential effects of the PAT results on national policy-making (e.g. by employing WHO’s PAT Dissemination Template, [53]) and on PA promotion action at the subnational and local level [56].

## Data Availability

Not applicable.

## Abbreviations

CAPPA: Comprehensive Analysis of Policy on Physical Activity
EU: European Union
GAPPA: Global Action Plan on Physical Activity
HEPA: Health-enhancing physical activity
NCD: Noncommunicable disease
NGO: Nongovernmental organization
NPAP: National Physical Activity Plan (Ireland)
PA: Physical activity
PEN: Policy Evaluation Network
PAT: Policy Audit Tool
